# Age-related lncRNA alterations of SDHAP2_miR-17-5p/miR-20b-5p_RAB11FIP1 ceRNA network in donor-derived human limbal epithelial cells

**DOI:** 10.3389/fcell.2026.1747752

**Published:** 2026-03-30

**Authors:** Shangkun Ou, Shaoxia Ye, Shengpeng Zhang, Sijie Lin, Yi Mao, Liying Zhang, Xiaodong Liu, Lingyu Zhang, Huping Wu, Yiming Wu

**Affiliations:** 1 Department of Ophthalmology, The Affiliated Hospital of Guizhou Medical University, Guiyang, Guizhou, China; 2 The Second Affiliated Hospital of Xiamen Medical College, Xiamen, China; 3 School of Medicine, Xiamen Eye Center and Eye Institute of Xiamen University, Xiamen, China; 4 Fujian Provincial Key Laboratory of Ophthalmology and Visual Science, Fujian Engineering and Research Center of Eye Regenerative Medicine, School of Medicine, Xiamen University, Xiamen, China; 5 National Clinical Research Center for Ocular Diseases, Eye Hospital, Wenzhou Medical University, Wenzhou, China; 6 National Engineering Research Center of Ophthalmology and Optometry, Eye Hospital, Wenzhou Medical University, Wenzhou, China; 7 State Key Laboratory of Ophthalmology, Optometry and Visual Science, Eye Hospital, Wenzhou Medical University, Wenzhou, China; 8 Department of Ophthalmology, The First Affiliated Hospital of Zhengzhou University, Zhengzhou, China; 9 Tianjin Key Laboratory of Retinal Functions and Diseases, Tianjin Branch of National Clinical Research Center for Ocular Disease, Eye Institute and School of Optometry, Tianjin Medical University Eye Hospital, Tianjin, China

**Keywords:** aging, ceRNA, corneal epithelium, limbal epithelial cells, lncRNA, microRNA, ocular surface, whole transcriptome sequencing

## Abstract

**Background:**

Limbal epithelial cells (LECs) play a crucial role in preserving ocular surface stability and ensuring the normal function of the corneal epithelium. The functional capacity of LECs diminishes with age, playing a part in the onset of ocular diseases linked to aging. Although long non-coding RNAs (lncRNAs) are key regulators of gene expression and are known to be involved in numerous ocular pathologies, their expression dynamics during aging in LECs are not yet well characterized.

**Methods:**

High-throughput RNA sequencing and computational analysis were utilized to characterize age-related differences in mRNA and lncRNA in human LECs derived from young and old donors. 90 lncRNAs and 177 mRNAs with significant age-associated expression changes were identified. Functional enrichment was assessed using Gene Ontology (GO) and Kyoto Encyclopedia of Genes and Genomes (KEGG) pathway analyses. A competing endogenous RNA (ceRNA) network was constructed using Cytoscape and cytoHubba, focusing on the interaction between lncRNAs, miRNAs and mRNAs.

**Results:**

The study identified the potential ceRNA network, SDHAP2_miR-17-5p/miR-20b-5p_RAB11FIP1, that might be crucial in age-related changes of the LECs. Quantitative RT-PCR validated the expression for SDHAP2 (downregulated), miR-17-5p (upregulated), miR-20b-5p (upregulated), and RAB11FIP1 (downregulated) in the old group, consistent with transcriptome data. Functional analysis suggested this network may be involved in oxidative stress responses and cellular senescence.

**Conclusion:**

Our findings reveal age-associated lncRNA and mRNA expression alterations in human LECs and highlight the SDHAP2_miR-17-5p/miR-20b-5p_RAB11FIP1 ceRNA network as a potential molecular indicator and therapeutic entry point for age-related ocular surface diseases.

## 1 Introduction

The integrity and homeostasis of the ocular surface, particularly the corneal epithelium, relies on limbal epithelial cells (LECs) (Li et al., 2007; Long et al., 2024; Zheng et al., 2025), and their dysfunction has been reported to be associated with oxidative stress and inflammatory responses (Dudok et al., 2015; Lin et al., 2023; Tian et al., 2024). They have likewise been implicated as potential drivers in age-associated ocular disorders such as meibomian gland dysfunction, diabetic keratopathy, keratoconus, cataract, pterygium, and dry eye disease (Bohm et al., 2023; Wiedemann et al., 2024; Khaled et al., 2018). Emerging evidence further indicates that aging is associated with structural and functional alterations in LECs. *In vivo* confocal microscopy revealed that the visibility of Vogt palisades in the human limbus diminishes with advancing age. Additionally, an increase in cell size associated with aging resembles morphological features typically observed in limbal basal epithelial cells (Zheng and Xu, 2008). Age-associated thinning of the paracentral corneal, nasal and temporal limbal epithelium was identified by Yang et al. using anterior segment optical coherence tomography to assess changes in epithelial thickness across different age groups (Yang et al., 2014). Consistently, Li et al. demonstrated by OCT that limbal epithelial thickness was negatively correlated with age (Li et al., 2022). Spectral-domain optical coherence tomography also revealed a reduced detection rate of typical Vogt’s palisades across the superior, nasal, temporal, and inferior limbal regions (Lin et al., 2016). Improved regenerative capacity has been observed in limbal epithelial stem cell sheets derived from donors under the age of 65, according to *in vitro* findings (Yang et al., 2022). A reduction in stem cell activity potentially associated with aging is further supported by the limited presence of corneal clones observed in mice (Douvaras et al., 2012).

As noncoding transcripts longer than 200 nucleotides, long non-coding RNAs (lncRNAs) lack protein-coding potential but exert significant influence over gene expression and decisions in cell fate determination (Chodurska and Kunej, 2025). lncRNAs contribute to fundamental biological events such as lineage commitment, cellular differentiation, organ formation, maintenance of tissue equilibrium, and the progression of various human disorders (Chini et al., 2024). Noncoding RNA (ncRNAs), including lncRNAs, circular RNAs, and pseudogenes, can function as competing endogenous RNAs (ceRNAs) or act as endogenous miRNA sponges. Through microRNA (miRNA) binding, they modulate the post-transcriptional regulation of miRNA target genes (Tay et al., 2014). The involvement of lncRNAs in multiple eye diseases has been well documented. In diabetic retinopathy, endothelial-mesenchymal transition is regulated, in part, by lncRNA H19 through specific molecular interactions (Thomas et al., 2019). Ischemia-reperfusion induces a significant increase in lncRNA H19 levels. This upregulation promotes NLRP3 and NLRP6 inflammasome imbalance, leading to overproduction of proinflammatory cytokines, microglial pyroptosis, and ultimately neuronal death. The regulatory function of lncRNA H19 in the ischemic cascade is mediated through its interaction with miR-21, which allows for upregulation of PDCD4 via a ceRNA mechanism (Wan et al., 2020). Emerging evidence suggests a regulatory role for lncRNAs in corneal neovascularization, indicating their potential as novel targets for therapeutic development (Huang et al., 2015; Bai et al., 2018; Sun et al., 2019; Liu et al., 2021; Shen et al., 2022). By analyzing keratoconus-affected corneal tissue, Khaled et al. revealed distinct expression patterns of coding genes and lncRNAs, indicating potential molecular involvement in disease progression (Khaled et al., 2018). A recent study employed integrative analysis of the ceRNA network to explore how lncRNAs are linked to inflammatory processes in mixed-type dry eye disease (Tang et al., 2022). While lncRNAs are known to play roles in ocular biology, age-associated expression changes in the human limbus have yet to be thoroughly investigated.

**FIGURE 1:**
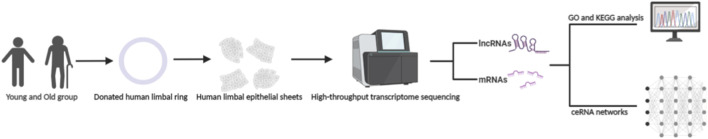
Diagrammatic sketch presenting the methods used in this study.

In an effort to further identify aging-related lncRNAs and investigate their functional mechanisms, the study conducted a transcriptome-wide comparison of mRNA and lncRNA expression between the young and old groups via high-throughput transcriptome sequencing (Figure 1). Analysis of aging-related lncRNA expression changes led to the identification of the SDHAP2_miR-17-5p/miR-20b-5p_RAB11FIP1 axis as a distinct ceRNA network. This study further performed RT-qPCR to confirm the differential expression patterns of selected lncRNAs. In summary, the SDHAP2_miR-17-5p/miR-20b-5p_RAB11FIP1 axis was identified as a potentially key ceRNA regulatory network in age-related ocular surface disorders, offering possible biomarker candidates and therapeutic avenues for conditions linked to oxidative damage.

## 2 Materials and methods

### 2.1 Isolation of human LECs

Human limbus tissue was obtained from corneal transplant specimens donated by the Human Ethics Committee of Xiamen University Affiliated Xiamen Eye Center. The information on cornea donors for whole transcriptome sequencing is summarized in Table 1. All experimental protocols adhered to the Declaration of Helsinki and the ARVO guidelines for research in ophthalmology and vision science, and were approved by the Ethics Committee of Xiamen University. Researchers dissected limbal tissue into fragments, which were subsequently incubated with 2 mg/mL dispase II in SHEM medium at 4 °C for 14 h. Limbal epithelial sheets were separated using iris restorer and toothless forceps under an ophthalmic surgery microscope (VISU150, Carl ZEISS, Germany). Limbal epithelial samples for whole transcriptome sequencing were obtained from five donors. An independent set of limbal epithelial samples from six donors was used for RT-qPCR validation.

**TABLE 1:** Donor information for the RNA sequencing cohort.

Donor	Age	Gender	Cause of death
Young-1	29	Female	Traffic accidents
Young-2	21	Male	Brain tumor with hemorrhage
Young-3	31	Male	Cerebral hemorrhage
Old-1	58	Male	Hemorrhage
Old-2	64	Female	Traffic accidents

### 2.2 H&E staining

Fresh human limbus rings from the donors were immediately fixed overnight in 4% paraformaldehyde, followed by embedding in OCT for histological analysis. Tissue sections of 7 μm thickness were stained using hematoxylin and eosin according to the manufacturer’s instructions and previous protocol (Zhang et al., 2022), and subsequently examined with a Zeiss light microscope.

### 2.3 Whole transcriptome sequencing

The young (Age 21, Age 29, and Age 31) and old (Age 58 and Age 64) groups of human limbal epithelial sheets were obtained as described above. TRIzol reagent (T1129131; Yeasen, Shanghai, China) was used for RNA extraction in accordance with the supplier’s instructions. rRNA was selectively eliminated from total RNA preparations with the aid of the MGIEasy rRNA removal kit. After constructing the RNA libraries, the DNBSEQ platform (Beijing Genomics Institute, BGI, China) was used for sequencing.

Before downstream analysis, raw sequencing data were subjected to quality control to eliminate reads with low sequencing quality, adaptor contamination, and excessively high levels of unknown nucleotides (N). After filtering, each sample retained approximately 111 million clean reads. More than 94.9 percent of reads were successfully mapped to the reference genome and 87 to 90 percent were uniquely mapped, indicating high sequencing depth and reliable mapping performance. The detailed statistics of the filtered sequencing reads are provided in Table 2.

**TABLE 2:** Reads filtering.

Donor	Total clean reads (M)	Total mapping (%)	Uniquely mapping (%)
Young-1 (age 29)	111.46	95.86	88.92
Young-2 (age 21)	111.52	95.92	89.49
Young-3 (age 31)	111.19	94.94	88.6
Old-1 (age 58)	111.37	95.21	87.42
Old-2 (age 64)	111.54	95.97	89.91

Following genome alignment, alternative splicing events were detected using rMATS. Five categories of alternative splicing were identified, including skipped exon, alternative 5′splicing site, alternative 3′splicing site, mutually exclusive exons, and retained intron. The splicing event counts were generated as a technical output of the sequencing pipeline to provide an overview of transcript splicing diversity in each donor sample. The detailed statistics of these five splicing categories are presented in Table 3.

**TABLE 3:** Alternative splicing.

Donor	Alternative 5′splicing site	Alternative 3′splicing site	Mutually exclusive exons	Retained intron	Skipped exon	Total
Young-1 (age 29)	3,339	4,024	5,068	1,412	29,078	42,921
Young-2 (age 21)	3,480	4,293	6,009	1,442	33,085	48,309
Young-3 (age 31)	3,318	3,926	4,769	1,344	28,717	42,074
Old-1 (age 58)	3,172	3,720	3,165	1,337	20,470	31,864
Old-2 (age 64)	3,240	3,987	4,737	1,363	28,128	41,455

### 2.4 Construction of ceRNA network

miRNA-binding sites within ncRNAs and mRNAs were identified through miRanda and TargetScan algorithms. The resulting differentially expressed (DE) miRNAs were mapped to their respective lncRNA and mRNA targets to infer ceRNA network interactions.

### 2.5 Gene Ontology enrichment and KEGG pathway analysis

Functional annotation of genes related to age-associated ocular disorders was performed via Gene Ontology (GO) analysis using the Dr. Tom platform (BGI) in conjunction with data from the official GO database. Identified mRNAs were classified under molecular functions (MFs), cellular components (CCs), and biological processes (BPs). Pathway enrichment was conducted using the Kyoto Encyclopedia of Genes and Genomes database, and results were visualized in the form of scatter plots.

### 2.6 Real-time quantitative PCR

Real-time quantitative PCR validation was performed using limbal epithelial samples from six independent donors (three young and three old), distinct from those used for RNA sequencing. Total RNA was isolated from both young and old groups using TRIzol reagent (T1129131; Yeasen, Shanghai, China) in accordance with the manufacturer’s guidelines. For miRNA analysis, reverse transcription and subsequent PCR amplification were conducted with the miRNA qPCR Quantitation Kit (GenePharma, Shanghai, China), employing U6 as the internal reference. Other cDNA was synthesized using the HiScript II Reverse Transcriptase kit (R201-01; Vazyme, Nanjing, China). Quantitative PCR amplification was carried out on a Roche LightCycler 96 system using SYBR Premix Ex Taq (Takara). Each donor sample was considered a biological replicate, and the young and old groups consisted of three biological replicates each. For each biological replicate, qPCR was performed in triplicate wells, which were treated as technical replicates. GAPDH and U6 served as internal reference genes, and relative gene expression levels were calculated using the 2^-ΔΔCT^ method. The specific primer sequences used for amplification are provided in Table 4.

**TABLE 4:** Primer sequences used in the present study.

Gene symbol	Forward primer	Reverse primer
SDHAP2	CAG​TCA​AGG​CGA​AAG​GTT​TAT​G	TGC​TCT​GGA​GGT​AGG​TGG​TG
miR-17-5p	ATT​CTT​CCA​AAG​TGC​TTA​CAG​TGC	TAT​GGT​TTT​GAC​GAC​TGT​GTG​AT
miR-20b-5p	AGC​ATA​CAA​AGT​GCT​CAT​AGT​GC	TAT​GGT​TTT​GAC​GAC​TGT​GTG​AT
RAB11FIP1	GGA​CAA​GGA​GCG​AGG​AGA​AAT	GTC​GTG​CTA​GGG​ATG​ATG​GC
GAPDH	ACA​ACT​TTG​GTA​TCG​TGG​AAG​G	GCC​ATC​ACG​CCA​CAG​TTT​C
U6	GCT​TCG​GCA​GCA​CAT​ATA​CTA​AAA​T	CGC​TTC​ACG​AAT​TTG​CGT​GTC​AT

### 2.7 Statistical analysis

Statistical analysis was conducted using SPSS version 22.0. Comparisons between groups were assessed using Student’s t-test, as appropriate. Data are expressed as mean ± standard deviation (SD), with p < 0.05 considered statistically significant.

## 3 Results

### 3.1 Age-related changes of human limbal epithelial cells

**FIGURE 2:**
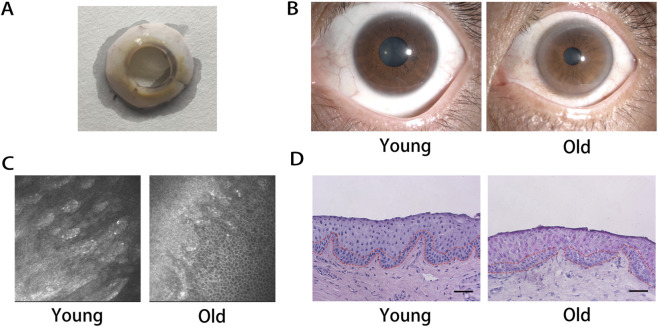
Age-related changes of human limbal epithelium. **(A)** Human limbal ring was obtained from corneal transplant donors. Slit lamp microscope **(B)** and *in vivo* confocal microscopy **(C)** of healthy volunteers. **(D)** H&E staining of donated human limbus. Red dotted box: human limbal epithelial basal cells. Scale bar represents 50 μm.

Age has been shown to affect multiple corneal properties including elasticity (Knox Cartwright et al., 2011), refraction (Shao et al., 2017), transparency (Consejo et al., 2021), and thickness (Colakoglu and Cosar, 2022). As corneal tissues age, structural and functional decline may predispose individuals to dry eye disease, characterized by glandular dysfunction, tear film disruption, and progressive visual disturbance (Gipson, 2013). The age-related alterations in the limbal region of healthy individuals were investigated by slit-lamp and *in vivo* confocal microscopy. As illustrated in Figures 2A,B, the old group exhibited a notable reduction in corneal transparency compared to the young group. The limbal stem cell niche, situated within the palisades of Vogt, was clearly identifiable in young individuals under *in vivo* confocal microscopy, but appeared structurally diminished and less frequent in older subjects (Figure 2C). The results of H&E staining (Figure 2D) were consistent with those of confocal microscopy, in addition to the enlargement and decreased density of basal cells in the old group.

### 3.2 Differentially expressed lncRNAs and mRNAs

**FIGURE 3:**
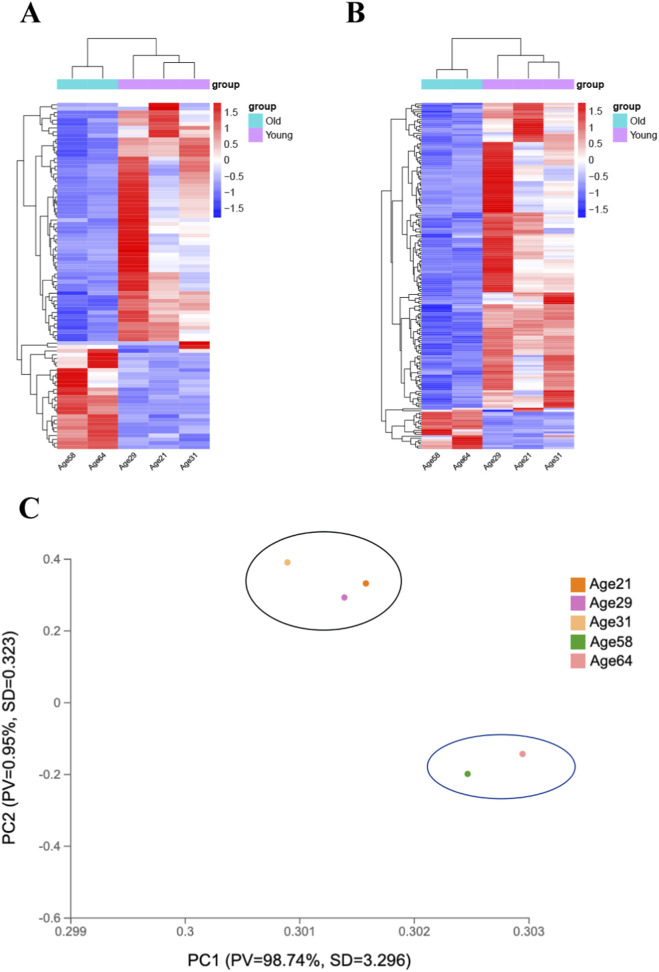
Expression profiles of lncRNAs and mRNAs. Heatmaps clustering of all significantly differentially expressed lncRNAs **(A)** and mRNAs **(B)** in human limbal epithelium sheets of young (Age 21, Age 29, Age 31) and old group (Age 58, Age 64). **(C)** PCA plot showing distinct transcriptomic separation between the two groups.

Expression profiling of lncRNAs and mRNAs was conducted in human limbal epithelium samples from young and old individuals. Differential expression was determined using a fold change cutoff of 1.0 together with a Q value ≤0.05 to capture moderate age-associated transcriptomic shifts. The Q value represents the false discovery rate adjusted p value. Based on these filtering criteria, a total of 90 lncRNAs (27 upregulated and 63 downregulated) and 177 mRNAs (19 upregulated and 158 downregulated) were identified as significantly DE. Hierarchical clustering revealed clear transcriptomic distinctions between the two age groups (Figures 3A,B). In addition, principal component analysis further confirmed the separation of the two groups at the global transcriptomic level (Figure 3C). The full lists of DE lncRNAs and mRNAs are provided in Supplementary Tables S1, S2.

### 3.3 Gene Ontology enrichment and KEGG pathway analysis

**FIGURE 4:**
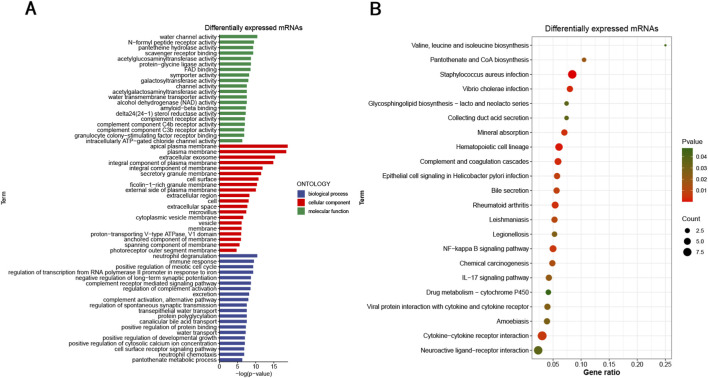
GO and KEGG pathway analysis of DE mRNAs. **(A)** Top 20 terms of GO enrichment analysis. **(B)** Top 22 KEGG enriched pathways.

Key regulators and biological pathways implicated in age-related transcriptomic changes were identified through GO enrichment of DE mRNAs. Figure 4A displays the top 20 enriched terms under the BPs, CCs, and MFs categories. Notably, several of these terms are closely linked to aging of the ocular surface. For instance, “water channel activity”, “plasma membrane”, and “transepithelial water transport” are associated with aquaporins (AQPs), which are involved in maintaining corneal transparency and corneal epithelial repair (Verkman et al., 2008). Furthermore, the enrichment of BPs such as “positive regulation of meiotic cell cycle” and “positive regulation of developmental growth” reflects significant alterations in cell proliferation-related signatures, suggesting a shift in the regenerative capacity of the limbal epithelium during aging.

KEGG pathway analysis was employed to functionally classify DE mRNAs. The 22 top-ranking KEGG pathways associated with distinct categories of DE mRNAs were visualized in Figure 4B. A substantial portion of the KEGG pathways linked to DE mRNAs were related to viral and bacterial infections, highlighting enrichment in pathways associated with maintaining corneal epithelial barrier integrity (Leong and Tong, 2015). Additionally, the enriched term “NF-kappa B signaling pathway” has been reported to function in TGF-β signaling pathway crosstalk, which has been previously linked to RNA stress-mediated promotion of corneal epithelial senescence (Li et al., 2016). Notably, the enrichment of “Chemical carcinogenesis” and “Cytokine-cytokine receptor interaction”, along with the NF-kappa B pathway, further underscores the modulation of cell proliferation and inflammatory signaling, which are hallmarks of the functional decline in aged limbal tissues.

### 3.4 Construction and analyses of ceRNA networks

lncRNAs modulate gene expression by sequestering miRNAs, thereby regulating downstream mRNA targets through ceRNA competition. To examine this process in the context of aging, ceRNA networks were established using DE transcripts and predicted via TargetScan, MiRanda, and MiRTarBase.

**FIGURE 5:**
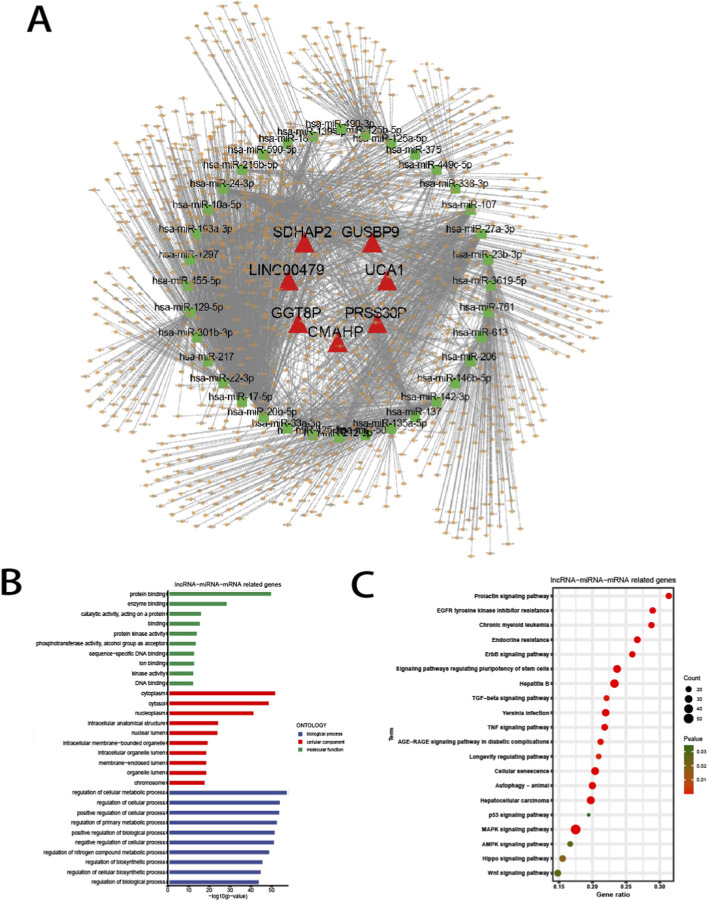
Construction of the lncRNA-miRNA-mRNA Network. **(A)** Network analysis of lncRNA (triangle)-miRNA (square)-mRNA (round). **(B)** Top 10 GO terms analyzed with mRNAs in lncRNA-miRNA-mRNA network. **(C)** Top 20 KEGG pathway enrichment of mRNAs in lncRNA-miRNA-mRNA network.

As illustrated in Figure 5A, seven lncRNAs were identified as potential miRNA sponges, contributing to the construction of 36 distinct ceRNA networks encompassing 1,165 miRNA target genes. To gain further insight into the functional roles of these networks, GO and KEGG pathway analyses were conducted on the predicted targets (Figure 5B). The top MF terms primarily involved “protein binding” and “enzyme activity,” mirroring the GO enrichment trends observed in the differentially expressed mRNAs. The top BPs terms, including “regulation of cellular metabolic process”, “regulation of cellular process” and “regulation of biosynthetic process” are widely involved in cell senescence. The enriched terms of KEGG pathways “Autophagy,” “Cellular senescence,” and “Longevity regulating pathway” are closely associated with aging of corneal epithelium. In addition, the term “TGF-beta signaling pathway” has been reported to activate the “NF-kappa B signaling pathway” during corneal epithelial senescence and was concurrently highlighted in KEGG enrichment results based on the DE mRNA dataset (Figure 5C) (Li et al., 2016).

### 3.5 Validation of associated lncRNA, miRNA, and mRNA expression levels in the ceRNA network between the young and old groups

**FIGURE 6:**
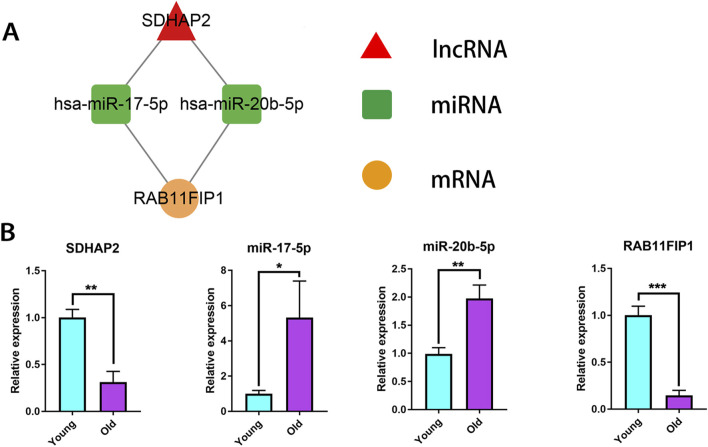
Validation of associated lncRNAs, miRNAs and mRNA expression levels. **(A)** Overlapped ceRNA network between predicted and sequencing results. **(B)** Relative expression of SDHAP2, miR-17-5p, miR-20b-5p and RAB11FIP1 via RT-qPCR test. Data represent mean ± SD. *p < 0.05, **p < 0.01, ***p < 0.001. Young: n = 3 donors; Old: n = 3 donors. Each donor was measured in triplicate.

To investigate the key lncRNAs in the aging of limbal epithelium, the predicted ceRNA networks were overlapped with the DE lncRNAs and mRNAs. As shown in Figure 6A, SDHAP2_miR-17-5p/miR-20b-5p_RAB11FIP1 was considered a potential ceRNA network. RT-qPCR was used to assess the expression patterns of the selected lncRNAs, miRNAs, and mRNAs involved in the ceRNA network in an independent cohort of donors (Figure 6B). In the Old group, SDHAP2 expression was markedly reduced, while miR-17-5p and miR-20b-5p levels were elevated. Their upregulation coincided with reduced RAB11FIP1 expression. The qRT-PCR results for SDHAP2 and RAB11FIP1 showed age-associated expression trends that were consistent with the transcriptomic data obtained from high-throughput sequencing. The involvement of miR-17-5p and miR-20b-5p in various ocular pathologies has been documented in previous studies. For example, angiogenesis in retinopathy of prematurity may be influenced by miR-17-5p and miR-20a-5p through their regulatory effects on HIF-1α and VEGF signaling pathways (Guo et al., 2021). In diabetic retinas, reduced expression of circDNMT3B has been implicated in vascular abnormalities through its modulation of miR-20b-5p and BAMBI (Zhu et al., 2019). RAB11FIP1 belongs to the Rab11-interacting protein family (Rab11-FIPs) and has been proposed as a therapeutic candidate in the context of cervical cancer (Zhang et al., 2021). However, the involvement of lncRNAs, miRNAs, and mRNAs within this ceRNA network remains uninvestigated in the context of human corneal biology. Therefore, SDHAP2_miR-17-5p/miR-20b-5p_RAB11FIP1 was identified as a distinct ceRNA regulatory axis characterized by age-associated expression patterns in human limbal epithelium.

## 4 Discussion

Aging is a multifactorial biological process often linked to oxidative stress, a major contributor to the onset of numerous diseases (Shen et al., 2024). Accumulating evidence indicates that age can significantly impact the ocular surface, including structural and functional alterations in the cornea (Gipson, 2013; De Silva et al., 2019). The integrity and renewal capacity of the corneal epithelium are primarily maintained by LECs residing in specialized limbal niches (Li et al., 2007). Several investigations have documented aging-associated modifications in the human limbal region (Zheng and Xu, 2008; Yang et al., 2014; Yang et al., 2022). However, the association between aging and the human limbal system has not been elucidated in detail, and the underlying mechanism remains unclear.

ncRNAs are involved in a wide range of cellular functions and have been associated with numerous disease states. ncRNAs encompass both short transcripts such as miRNAs and longer species including circular RNAs, pseudogenes, antisense RNAs and lincRNAs. These molecules have been widely implicated in post-transcriptional gene regulation through ceRNA networks, in which lncRNAs function as molecular sponges for miRNAs, thereby influencing the expression of target genes (Chan and Tay, 2018). Numerous studies have shown that lncRNAs and miRNAs play crucial regulatory roles in the pathogenesis of a range of eye disorders, including diabetic retinopathy (Thomas et al., 2019), corneal neovascularization (Huang et al., 2015), human corneal endothelial dysfunction under oxidative stress (Shan et al., 2021), keratoconus (Khaled et al., 2018), and dry eye (Tang et al., 2022). However, little is known about the expression patterns of lncRNAs in the human limbal epithelium, let alone how they change with age. Moreover, although many studies (Collin et al., 2021; Davidson et al., 2024; Altshuler et al., 2021) have used various transcriptomic sequencing approaches to investigate the human limbal epithelium and have to some extent revealed age-related changes, they have not specifically examined lncRNAs. In the present study, human limbal epithelial sheets were isolated from donated limbus rings. High-throughput transcriptome sequencing was conducted to compare DE lncRNAs and mRNAs between the young and old groups. Our results showed that 90 lncRNAs (27 upregulated and 63 downregulated) and 177 mRNAs (19 upregulated and 158 downregulated) were DE.

The functions of DE lncRNAs and mRNAs in the old group were predicted using GO enrichment (BP, CC, and MF) and KEGG pathway analyses. Among the enriched terms of BP, CC, and MF, “water channel activity,” “water transmembrane transporter activity,” “integral component of plasma membrane,” and “transepithelial water transport” were highly associated with AQPs. AQPs constitute a group of transmembrane channel proteins that facilitate the transport of water and small solutes across cellular membranes, and they are evolutionarily conserved across animals, plants, and bacteria. Among them, AQP5 is notably expressed in both corneal epithelial cells and stromal keratocytes of the human eye. It plays a critical role in fluid movement from the corneal stroma to the tear film, contributing to osmotic balance. Notably, mice lacking AQP5 exhibit elevated tear fluid osmolality, indicating its importance in tear homeostasis (Ruiz-Ederra et al., 2009). RNA-sequencing revealed a marked reduction in AQP5 expression in old samples, which may be relevant to previously reported findings of compromised ocular surface integrity and tear film instability in systematic reviews and meta-analyses examining age-related dry eye pathophysiology (Kitazawa et al., 2022). Among the top20 enriched terms of KEGG pathways, most of them are closely related to aging, such as “EGFR tyrosine kinase inhibitor resistance,” “ErbB signaling pathway,” “TNF signaling pathway,” “Longevity regulating pathway” and “Cellular senescence” etc. Interestingly, the “NF-kappa B signaling pathway” and “TGF−beta signaling pathway” were enriched in DE mRNAs and lncRNA-miRNA-mRNA-related genes, respectively. Elevated expression of TGF-β, along with senescence markers p16 and p21, has been observed in the corneal epithelium of older donors. Moreover, activation of the NF-κB pathway by TGF-β has been linked to corneal epithelial aging through RNA metabolic dysregulation, while anti-inflammatory interventions have been shown to mitigate TGF-β-induced senescent changes (Li et al., 2016).

After construction of the ceRNA networks, the lncRNA-related genes were overlapped with the DE mRNAs, and SDHAP2_miR-17-5p/miR-20b-5p_RAB11FIP1 was identified as a unique ceRNA network. In the older group, SDHAP2 expression was markedly reduced, along with decreased levels of RAB11FIP1. In contrast, miR-17-5p and miR-20b-5p showed significant upregulation. In fact, the downregulation of miR-17-5p has been observed in many aging tissues (such as the heart) (Dellago et al., 2017) and aging cells (such as fibroblasts) (Zhuo de et al., 2010). However, some exceptions have been reported in the mouse aging brain, where both miR-17-5p and miR-17-3p (formerly annotated as mmu-miR-17 and mmu-miR-17*, with the old “*” tag not fixedly mapping to 5p or 3p) were found to be upregulated in aged mouse samples (Inukai et al., 2012). The expression trend of miR-20b-5p in aging tissues is also controversial, being downregulated in the aging thymus (Guo et al., 2017) and aging retina (Hermenean et al., 2020) of mice, but upregulated in vascular aging induced by hyperhomocysteinemia (Qin et al., 2023). Notably, miR-17-5p and miR-20b-5p are well-recognized regulators of the cell cycle. Specifically, miR-17-5p has been identified as a key driver of the G1/S phase transition by coordinating the suppression of negative regulators (Cloonan et al., 2008). Similarly, miR-20b-5p has been shown to inhibit cell cycle progression and proliferation by targeting the CCND1/CDK4/FOXM1 axis in other cell types (Yang et al., 2020). In our identified ceRNA network, the age-related upregulation of these miRNAs might disrupt the fine-tuned cell cycle regulation in LECs, potentially contributing to the diminished regenerative capacity and proliferative exhaustion characteristic of the aging limbal epithelium.

RAB11FIP1, belonging to the Rab11-interacting protein family, contributes to the regulation of vesicle transport and epithelial cell polarity via its interaction with the small GTPase Rab11a (Novick and Zerial, 1997). Rab11FIP1 deficiency in mice has been associated with mucosal goblet cell loss, spontaneous inflammatory responses and heightened vulnerability to colonic injury (Rathan-Kumar et al., 2022). Moreover, reduced expression of Rab11FIP1 has been identified in the immune microenvironment of lung adenocarcinoma, leading to altered infiltration levels of dendritic cells, neutrophils, macrophages, CD4 T cells, CD8 T cells, and B cells (Zhang et al., 2021). A separate study reported that depletion of Rab11FIP1 in human esophageal squamous cell carcinoma cell lines led to reduced E-cadherin expression and elevated mesenchymal lineage markers, indicating a potential induction of epithelial-mesenchymal transition (Tang et al., 2021). Interestingly, a significant decline in Rab11FIP1 expression was observed in the old group, which may be associated with the increased inflammation and immune alterations associated with the aging of the human cornea. Additional evidence indicates that Rab11FIP1 participates in the regulation of intercellular protein transport (Carson et al., 2013; Iannantuono and Emery, 2021; Fan et al., 2021), which may also partially explain functional impairments in the aging cornea. However, direct evidence linking Rab11FIP1 to tissue aging has not yet been thoroughly reported.

Although transcriptome sequencing revealed the potential involvement of the SDHAP2_miR-17-5p/miR-20b-5p_RAB11FIP1 ceRNA network in age-related lncRNA alterations in LECs, it is important to note that the current findings are primarily predictive and descriptive. Further elucidation of the underlying mechanisms will require direct functional validation through gain- and loss-of-function experiments, including siRNA knockdown and overexpression studies, including both *in vitro* and *in vivo* approaches. A significant challenge in investigating SDHAP2 arises from its status as a pseudogene, characterized by high sequence homology with its paralog SDHAP1 and the functional genes SDHA/SDHB. This extensive similarity poses a substantial hurdle for designing isoform-specific primers. While the primers employed in this study provided preliminary evidence of differential expression, we acknowledge the inherent difficulty in achieving absolute specificity. To ensure the reliability of such findings, future studies should incorporate rigorous specificity validations, such as Sanger sequencing of PCR products, to confirm the identity of the amplified sequences. Nonetheless, when integrated with our transcriptome sequencing data, the qPCR results provide supportive evidence for the involvement of SDHAP2 in the ceRNA network. In addition, miR-17-5p and miR-20b-5p share the conserved AAAGUGC seed region, implying partial overlap in their regulatory spectra (Haig and Mainieri, 2020). Such similarity may introduce a degree of redundancy into the ceRNA interactions involving these two miRNAs. Another limitation is the small number of donor-derived human limbal epithelial samples used for RNA sequencing (n = 3 for Young and n = 2 for Old), which reflects the inherent difficulty of obtaining healthy donor tissues rather than a design choice. To partially compensate for the limited number of sequencing samples, RT-qPCR validation was performed using a separate cohort of six donors (n = 3 for Young and n = 3 for Old), which provides an independent assessment of age-associated expression trends. Furthermore, the limbal epithelial sheets used in this study comprise a heterogeneous cell population, including both limbal stem/progenitor cells and differentiated epithelial cells. Therefore, the transcriptomic profiles captured here represent age-associated alterations at the tissue level rather than changes specific to a purified limbal stem cell population. Although cellular heterogeneity may dilute expression differences among individual subpopulations, it also reflects the biological reality that the limbal stem cell niche functions as an integrated epithelial system *in vivo*, where deterioration of the epithelial microenvironment is regarded as a major contributor to reduced regenerative capacity of the ocular surface. Nevertheless, the consistent transcriptomic clustering, the agreement between sequencing and qPCR, and the coherent ceRNA regulatory patterns together support the biological relevance of the identified SDHAP2_miR-17-5p/miR-20b-5p_RAB11FIP1 axis. The inherent heterogeneity of bulk tissue remains a challenge in fully capturing the cell-specific dynamics of this pathway. Consequently, our findings should be interpreted with caution regarding cell-type-specific contributions. Future investigations incorporating single-cell transcriptomics (Ou et al., 2025) are warranted to deconvolve these complex signals, thereby improving the robustness and clinical relevance of the proposed regulatory mechanism.

## 5 Conclusion

In conclusion, the study compared age-related lncRNA and mRNA changes in human LECs and identified ceRNA networks during aging. Our findings suggest that SDHAP2_miR-17-5p/miR-20b-5p_RAB11FIP1 could represent a ceRNA network associated with limbal epithelial senescence and may serve as a candidate for further biomarker exploration. By focusing on lncRNA expression, our findings contribute to a deeper understanding of limbal epithelial aging and may provide a molecular basis for future therapeutic research on age-related eye diseases.

## Data Availability

The original contributions presented in the study are included in the article/Supplementary Material, further inquiries can be directed to the corresponding author. The raw sequence data reported in this paper have been deposited in the Genome Sequence Archive (Zhang et al. 2025) in National Genomics Data Center (Members et al. 2025), China National Center for Bioinformation / Beijing Institute of Genomics, Chinese Academy of Sciences that are publicly accessible at https://db.cngb.org/data_resources/project/CNP0009062/.
